# In vitro antibacterial and anti-biofilm potential of an endophytic *Schizophyllum commune*

**DOI:** 10.1186/s13568-024-01663-x

**Published:** 2024-01-20

**Authors:** Avinash Sharma, Muzamil Rashid, Pooja Chauhan, Sukhraj Kaur, Amarjeet Kaur

**Affiliations:** Department of Microbiology, Guru Nanak Dev University, Amritsar, 143005 Punjab India

**Keywords:** *Schizophyllum commune*, Basidiomycetes, Antibacterial, Anti-biofilm, Endophyte

## Abstract

The emergence of antibiotic resistance in pathogens is one of the major health concerns facing mankind as different bacterial strains have developed resistance to antibiotics over the period of time due to overuse and misuse of antibiotics. Besides this, ability to form biofilms is another major factor contributing to antibiotic resistance, which has necessitated the need for exploration for novel and effective compounds with ability to inhibit biofilm formation. Endophytic fungi are reported to exhibit antibacterial and anti-biofilm potential and could serve as a potent source of novel antibacterial compounds. Majority of the bioactivities have been reported from fungi belonging to phylum Ascomycota. Endophytic basidiomycetes, inspite of their profound ability to serve as a source of bioactive compounds have not been exploited extensively. In present study, an attempt was made to assess the antibacterial, anti-biofilm and biofilm dispersion potential of an endophytic basidiomycetous fungus *Schizophyllum commune* procured from the culture collection of our lab. Ethyl acetate extract of *S. commune* showed good antibacterial activity against *Staphylococcus aureus*, *Klebsiella pneumoniae*, *Escherichia coli*, *Pseudomonas aeruginosa*, *Salmonella enterica* and *Vibrio cholerae*. Minimum inhibitory concentration and minimum bactericidal concentration of the extract were in the range of 1.25-10 mg/ml against the tested bacterial pathogens. The mode of action was determined to be bactericidal which was further confirmed by time kill studies. Good anti-biofilm activity of *S. commune* extract was recorded against *K. pneumoniae* and *S. enterica*, which was further validated by fluorescence microscopy. The present study highlights the importance of endophytic basidiomycetes as source of therapeutic compounds.

## Introduction

Development of resistance to antibiotics is one of the major threats to humanity in 21st century. Over the period of time different strains of bacteria have been reported to develop resistance against various antibiotics. There are several reasons which are responsible for antibiotic resistance in bacteria besides mutation of bacterial DNA through the course of evolution. The main reasons for antibiotic resistance are the overuse of antibiotics in agriculture sector and misuse with improper prescription in health sector (López Romo and Quirós 2019; Pulingam et al. 2022). Ability to form biofilms by pathogenic bacteria is another major cause of antibiotic resistance. Biofilm formation is the survival mechanism of microorganisms. These are sessile microbial communities embedded in self secreted extracellular polymeric substances (EPS) adhering to surface and/or with each other. EPS is composed of polysaccharides, proteins and DNA. Bacterial biofilms pose a major global health threat because of their potential to withstand the antibiotics, host immune system and various other external stresses. These properties of biofilms are responsible for the persistent chronic infection of pathogens and antimicrobial resistance (Flemming et al. 2016; Sharma et al. 2019; Vestby et al. 2020). According to Centers for Disease Control and Prevention, in United States more than 2.8 million people are infected with antibiotic resistant microbes every year and over 35,000 die because of this (CDC 2019). Worldwide these findings are more scary as about 0.7 million people die each year because of drug resistant microbes and the number of deaths is expected to increase to 10 million by 2050 (Kaur et al. 2021). Advancement of various medical practices also depends upon the antimicrobial efficacy. Different surgical and immunosuppressive treatments rely on antibiotic prophylaxis and their potency to treat complications related to infection. Thus, antibiotic resistance poses a major threat to health care system more than we assesse (MacGowan and Macnaughton 2017), which necessitates the exploration of novel and effective antimicrobial compounds. Recently, endophytes are gaining the attention of researchers as a source of natural antimicrobial compounds. Endophytes are the microorganisms which live within the living plant tissue without causing any symptomatic infection (Wilson 1995). Endophytic fungi isolated from different plant sources have been reported in several studies to exhibit good antimicrobial activity (Deshmukh et al. 2015, 2022; Farhat et al. 2019; Mbekou et al. 2021). Thus, endophytic fungi could serve as a prolific source of antimicrobial compounds. A survey of literature revealed that the majority of the endophytic fungi screened for antimicrobial potential belong to phylum Ascomycota. Ascomycota represent 84% of the total isolated endophytic fungi with Basidiomycota, Mucoromycota and Oomycota accounting for 10%, 5% and 1%, respectively (Rana et al. 2019). Thus, other phyla due to lack of representation have not been explored for their bioactive potential. Basidiomycota is a diverse group of fungi reported to exhibit various bioactivities. Most of the bioactivities have been documented from the compounds and extracts derived from the fruiting bodies of basidiomycetes (Jiao et al. 2013; Ditamo et al. 2016; Kou et al. 2021). Inspite of immense potential to synthesize bioactive compounds, endophytic basidiomycetes have not been explored much for their bioactivities. In previous studies conducted in our lab, we have attempted to isolate endophytic basidiomycetes. An endophytic *Schizophyllum commune* (Sch1) has been isolated from *Aloe vera* (Sharma et al. 2021). *S. commune* is an edible mushroom, also known as split gill mushroom, belonging to phylum Basidiomycota. *S. commune* has been used traditionally as a therapeutic for several illnesses including headache, indigestion, intestinal pain, obesity, inflammation and rheumatism (Guzmán 2008; Kamalebo et al. 2018). Even though different bioactivities of *S. commune* are reported (Yim et al. 2013; Mayakrishnan et al. 2013; Arun et al. 2015; Du et al. 2017; Rustamova et al. 2020) detailed investigations on biofilm inhibitory and antibacterial potential have not been carried out. Keeping this in view, this study aimed to assess the antibacterial, anti-biofilm and biofilm dispersion potential of an endophytic *Schizophyllum commune* (Sch1).

## Materials and methods

### Microorganisms

The endophytic basidiomycetous culture *S. commune* (Sch1) isolated from *Aloe vera,* was procured from the culture collection of our lab (Sharma et al. 2021), and has been deposited in National Fungal Culture Collection of India (NFCCI), Agharkar Research Institute, Pune, India vide accession number NFCCI 4838. The following bacterial strains *Staphylococcus aureus* (NCIM 5718), *Klebsiella pneumoniae* (NCIM 5215), *Escherichia coli* (NCIM 5662), *Pseudomonas aeruginosa* (NCIM 2862), *Salmonella enterica* (MTCC 733) and *Vibrio cholerae* (MTCC 3906) were used to determine the antibacterial, biofilm inhibitory and biofilm dispersion potential. All the chemicals used in the study were purchased from Himedia, Mumbai, India, except where specifically mentioned.

### Production of ***S. commune*** (Sch1) extract

*S. commune* (Sch1) was freshly grown on potato dextrose agar plate and one mycelial plug of 8 mm diameter was cut from the periphery of activated culture with the help of sterile borer. Thereafter, mycelial plug was inoculated in 250 ml Erlenmeyer flasks containing 50 ml production medium (malt extract 2%, dextrose 2% and peptone 0.1%). Flasks were then incubated at 180 rpm for 10 days at 30℃. Following incubation, extraction of the metabolites was done by using ethyl acetate and concentrated by using rotary evaporator (BUCHI). Obtained extract was re-suspended in phosphate buffer saline (PBS) (pH 7.4) for further use (Sharma et al. 2021).

### Determination of antibacterial activity

The antibacterial potential of *S. commune* (Sch1) extract was assessed using agar well diffusion method (Kaur and Sharma 2015). Overnight grown indicator pathogenic strains were diluted to obtain optical density (OD_595_) of 0.1. The inoculum was spread on nutrient agar media plates and wells of 6 mm diameter were cut by using sterile well-borer. Thereafter, the wells were filled with 100 µl of filter sterilized *S. commune* (Sch1) extract. The plates were initially incubated at 4 °C for 4 h to allow the inhibitors to diffuse into the nutrient agar media and then further incubated at 37 °C for 24 h. After 24 h, plates were examined and the zone of inhibition was measured in millimetres (mm).

### Determination of minimum inhibitory concentration (MIC) and minimum bactericidal concentration (MBC)

MIC of *S. commune* (Sch1) extract against different bacterial pathogenic strains was determined by broth dilution method (Parvekar et al. 2020). In a 96-well microtitre plate, two-fold serial dilutions of extract were prepared in 100 µl autoclaved nutrient broth. Pathogenic strains were grown overnight and diluted with sterile nutrient broth to get an OD_595_ of 0.1. Five µl of the culture suspension was added in each well and the plates were then incubated at 37 °C for 24 h, and visually inspected for turbidity. The MIC was calculated as the reciprocal of the lowest concentration at which no turbidity was seen.

For MBC determination, MIC broth along with other higher concentrations which showed no visible growth was inoculated on the nutrient agar plates. Thereafter, plates were incubated at 37 °C for 24 h. Minimum concentration which showed no bacterial growth was considered MBC.

### Time kill studies

Time kill study was performed by following the protocol described by Joshi et al. (2010) with some modifications. Extract at MBC value was added to 1 ml aliquots of autoclaved nutrient broth. Thereafter, mid exponential phase (0.1 OD_595_) grown pathogenic strain (40 µl) was added to the aliquots and incubated at 37 °C. Hundred microliters of sample was collected from the aliquots at different time intervals (0, 1, 2 4, 6 and 8 h) and spread on nutrient agar plates. After this, plates were incubated for 24 h at 37 °C and viable cell counts were expressed as log_10_ CFU/ml. The experiment was performed in triplicates.

### Propidium iodide staining

Effect of *S. commune* (Sch1) extract on membrane integrity of tested bacterial pathogens was determined by using method described by Sharma et al. (2020) with slight modifications. Bacterial pathogens were grown till mid-log phase in nutrient broth medium at 37 °C. Thereafter, grown culture was centrifuged (10,000 rpm) for 10 min, washed and re-suspended in PBS to obtain the final concentration of 1 × 10^6^ log_10_CFU/ml. *S. commune* (Sch1) extract at a concentration of MBC was added to the suspension and incubated at 37 °C for time duration as determined in time kill assays for different pathogens, where complete or 99% killing was obtained. After incubation, cell suspension was centrifuged at 10,000 rpm for 10 min. The obtained pellet was dissolved in PBS and coincubated with propidium iodide (Sigma-Aldrich, St. Louis, USA) solution (10 µg/ml) in dark for 15 min at 4 °C. To observe the cells stained with propidium iodide, cell suspension (10 µl) was placed on a glass slide and fixed with flourmount solution (5 µl). The fixed cells were then covered with a coverslip and examined under a fluorescent microscope (Olympus BX-43).

### Determination of biofilm inhibitory potential

Biofilm inhibitory activity of *S. commune* (Sch1) extract was assessed by using method described by Kaur et al. (2018). *S. commune* (Sch1) extract was tested for anti-biofilm activity at sub-MIC values. In 96 well plate, 100 µl of autoclaved nutrient broth was added to each well along with 100 µl extract and 20 µl overnight grown indicator pathogenic bacterial culture (OD_595_ of 0.1). To allow biofilm formation in the wells, the microtiter plate was incubated at 37 °C for 48 h. After 48 h, the plate was gently washed three times with autoclaved distilled water to remove non-adherent cells. The adherent cells were fixed for 15 min in 200 µl methanol, thereafter wells were emptied and air dried. The fixed biofilm was stained for 5 min with 200 µl of 2% crystal violet, and the excess stain was removed by washing with distilled water. Glacial acetic acid (160 µl of 33%) was used to extract stain from adherent cells, and OD_595_ was determined using microtitre plate reader. The control wells contained filtered PBS instead of *S. commune* (Sch1) extract. The experiment was performed in triplicates. The percentage (%) inhibition was calculated using the following formula:

$$Inhibition\left( \% \right) = 100 - \frac{{OD{\text{ }}of{\text{ }}sample \times 100}}{{OD{\text{ }}of{\text{ }}control}}$$

### Effect of ***S. commune*** (Sch1) extract on preformed biofilms

Effect of *S. commune* (Sch1) extract at sub-MIC values on preformed biofilms of bacterial pathogens was assessed by using method described by Kaur et al. (2018) with some modifications. Bacterial biofilm was developed in a 96-well microtiter plate by adding 100 µl autoclaved nutrient broth along with 20 µl of overnight grown culture (OD_595_ of 0.1), followed by incubation at 37 °C for 48 h. Non adherent cells were removed after incubation by gentle pipetting without damaging the biofilm. Thereafter, 100 µl autoclaved nutrient broth along with 100 µl extract was added to each well. In control wells PBS was added instead of extract. The plates were incubated for 48 h at 37 °C. The experiment was performed in triplicate. After, incubation the formed biofilm was quantified as previously described in biofilm inhibitory potential section.

### Fluorescent microscopy of biofilms

Two ml overnight grown pathogen adjusted to 0.1 OD_595_, containing sub-MIC value of extract was added in a 6 well plate. A sterile glass cover slip was placed in the well (on which the pathogen forms biofilm) and plate was incubated at 37 °C for 48 h. Control was devoid of fungal extract. After incubation, medium from the each well was decanted carefully. To remove the non-adherent cells, coverslips were gently washed two times with autoclaved distilled water followed by fixation of adherent cells with methanol for 10 min. Thereafter, 1 ml acridine orange (10 µg/ml) was added and allowed to stain cells for 10 min in dark. Excess of the stain was removed by washing two times with autoclaved distilled water. Coverslips were dried properly, fixed on glass slides and observed under confocal microscope (Nikon Corporation, Japan).

## Results

Resistance to antibiotics is a growing concern in the management of microbial diseases, necessitating the need for new and safe antibiotics. Therefore, in the present study, *S. commune* (Sch1) extract was assessed for its antibacterial and anti-biofilm activities against various Gram positive and Gram negative pathogenic bacteria including *S. aureus*, *K. pneumoniae*, *E. coli*, *P. aeruginosa*, *S.enterica* and *V. cholerae*.

### Screening for antibacterial potential

*S. commune* (Sch1) extract was found to possess antibacterial activity against all the tested bacterial pathogens (Table 1).

**Table 1:** Antibacterial activity of *S. commune* (Sch1) extract against different bacterial pathogenic strains

S. no.	Bacterial pathogen	Zone of inhibition
1	*S. aureus*	S^+^
2	*V. cholerae*	S^+^
3	*P. aeruginosa*	S^+^
4	*S. enterica*	S^+^
5	*K. pneumoniae*	S
6	*E. coli*	S^+^

S^+^ zone of inhibition ≥ 15 mm

S zone of inhibition < 15 mm

### Determination of MIC and MBC

MIC and MBC of the *S. commune* (Sch1) extract were determined against various pathogens to assess the efficacy, and the nature of the activity whether it is bacteriostatic or bactericidal. If the determined MBC/MIC is > 4, the antimicrobial compound is considered bacteriostatic, whereas if the compound shows MBC/MIC ≤ 4 then it may be considered bactericidal.

As shown in Table 2, S. *aureus* and *V. cholerae* were found to be most sensitive, with MIC of 1.25 mg/ml followed by *P. aeruginosa*. MBC values of *S. commune* (Sch1) extract were also determined for various pathogens. Low MBC value of 2.5 mg/ml was observed for *S. aureus, V. cholerae* and *P. aeruginosa*, whereas in case of *K. pneumoniae* the MBC was found to be 10 mg/ml.

**Table 2:** MIC and MBC of *S. commune* (Sch1) extract against different bacterial pathogenic strains

S. no.	Bacterial pathogen	MIC (mg/ml)	MBC (mg/ml)
1	*S. aureus*	1.25	2.5
2	*V. cholerae*	1.25	2.5
3	*P. aeruginosa*	2.5	2.5
4	*S. enterica*	5	10
5	*K. pneumoniae*	10	10
6	*E. coli*	5	5

The observed MBC/MIC ratio in this study was ≤ 2 against all the bacterial pathogens indicating the bactericidal nature of the *S. commune* (Sch1) extract.

### Time kill studies

The kinetics of killing of bacterial pathogens was studied by time kill assay. This study has also been used to determine the bacteriostatic and bactericidal nature of the antimicrobial compounds. If the initial bacterial count decreases in the presence of antimicrobial compound by ≥ 3log_10_ CFU/ml, then the compound is considered as bactericidal; whereas reduction of < 3log_10_ CFU/ml indicates the bacteriostatic nature of the compound. Time kill studies were performed at the obtained MBC values against different bacterial pathogenic strains. Bacterial cells exposed to *S. commune* (Sch1) extract for different time intervals were plated on nutrient agar plates. Emerged colonies were counted and compared with the initial bacterial viable cell count.

**Fig. 1:**
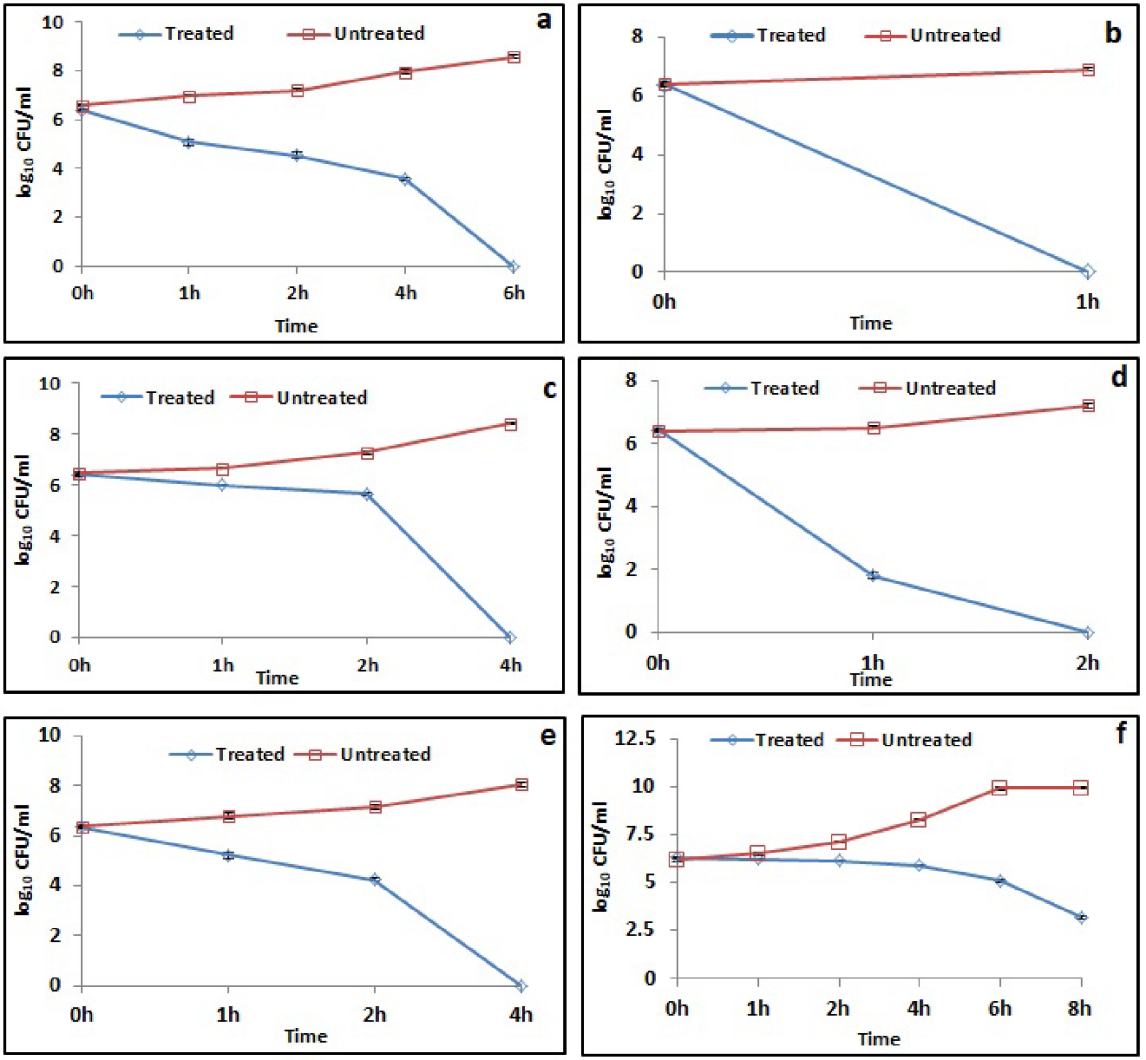
Time kill study against different bacterial pathogens in the presence of MBC of *S. commune* (Sch1) extract. **(a)**  *P. aeruginosa*; **(b)**  *S. enterica*; **(c)**  *E. coli*; **(d)**  *K. pneumoniae*; **(e)**  *V. cholerae*; **(f)**  *S. aureus*

As shown in Fig. 1a, untreated *P. aeruginosa* cells showed growth upto 8.57 ± 0.07 log_10_ CFU/ml after 6 h of incubation. On the other hand, exposure to *S. commune* (Sch1) extract resulted in complete killing of *P. aeruginosa* cells within 6 h. Treated cells showed a rapid decline in the viable cell count by 1.30, 1.84, 2.81 and 6.39 log_10_ CFU/ml after 1, 2, 4 and 6 h of incubation, respectively, as compared to the count at 0 h. Untreated *S. enterica* cells displayed an increase in growth from 6.40 ± 0.06 to 6.90 ± 0.05 log_10_ CFU/ml after 1 h of incubation. Whereas, treatment of *S. enterica* cells with *S. commune* (Sch1) extract caused complete killing with a decrease of 6.37 log_10_ CFU/ml viable cells, within 1 h of incubation (Fig. 1b). Similarly, in case of *E. coli* untreated cells displayed a rapid increase in the cell number from 6.45 ± 0.07 to 8.41 ± 0.04 log_10_ CFU/ml after 4 h of incubation. *S. commune* (Sch1) extract treated cells showed a decrease in the viable cell count by 0.39, 0.75 and 6.38 log_10_ CFU/ml after 1, 2 and 4 h of incubation, respectively, when compared to the initial viable cell number (Fig. 1c).

As shown in Fig. 1d, untreated *K. pneumoniae* cells showed growth from 6.39 ± 0.03 to 7.19 ± 0.06 log_10_ CFU/ml within 2 h of incubation. Exposure to *S. commune* (Sch1) extract at MBC value caused the complete killing of *K. pneumoniae* viable cells within 2 h of incubation. The viable counts showed a rapid decline of 4.62 and 6.41 log_10_ CFU/ml after 1 and 2 h of incubation, respectively, when compared with the count at 0 h. Untreated bacterial cells of *V. cholerae* showed growth up to 8.06 ± 0.09 log_10_ CFU/ml after 4 h of incubation from an initial count of 6.38 ± 0.05 log_10_ CFU/ml. Treatment of *V. cholerae* with *S. commune* (Sch1) extract displayed complete killing of viable cells within 4 h of incubation. The cell count showed a decrease of 1.12, 2.08 and 6.34 log_10_ CFU/ml after 1, 2 and 4 h of incubation, respectively, when compared to the initial viable cell count (Fig. 1e). Untreated *S. aureus* cells displayed maximum growth of 9.91 ± 0.04 log_10_ CFU/ml after 8 h of incubation whereas the cells treated with *S. commune* (Sch1) extract showed a gradual decrease in the bacterial number. Maximum decrease of 3.10 log_10_ CFU/ml, was observed at 8 h (Fig. 1f).

In this study, more than 3 log_10_ CFU/ml decrease in viable cells was observed in all the bacterial pathogens exposed to *S. commune* (Sch1) extract when compared with the initial viable cell count, revealing the bactericidal nature of the extract.

### Fluorescent microscopy of propidium iodide stained cells

**Fig. 2:**
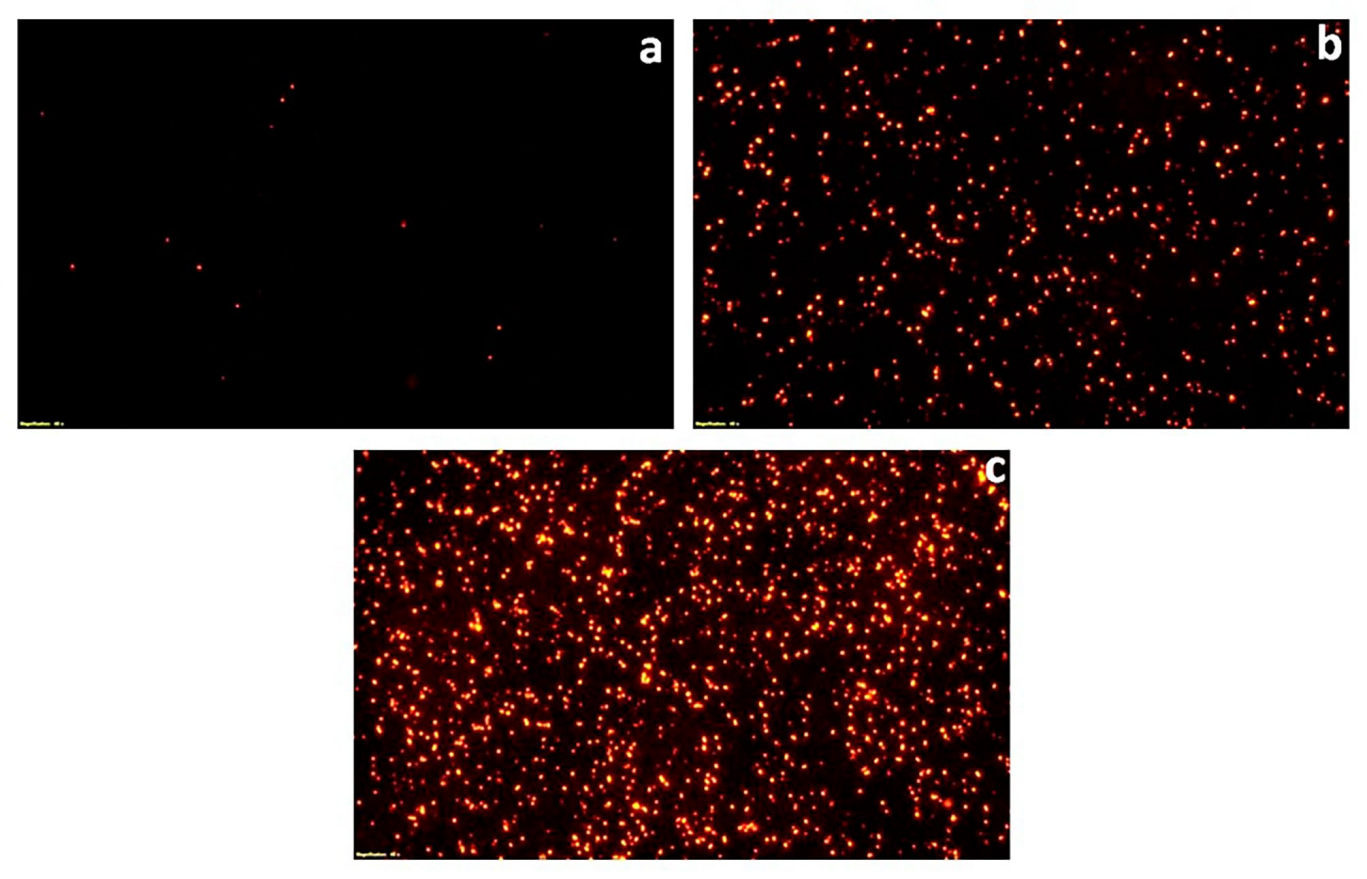
Fluorescent microscopic images of propidium iodide-stained *S. commune* (Sch1) extract treated/untreated *S. aureus* under 40X. **(a)** untreated; **(b)**  *S. commune* (Sch1) extract treated cells after 4 h **(c)**  *S. commune* (Sch1) extract treated cells after 8 h

**Fig. 3:**
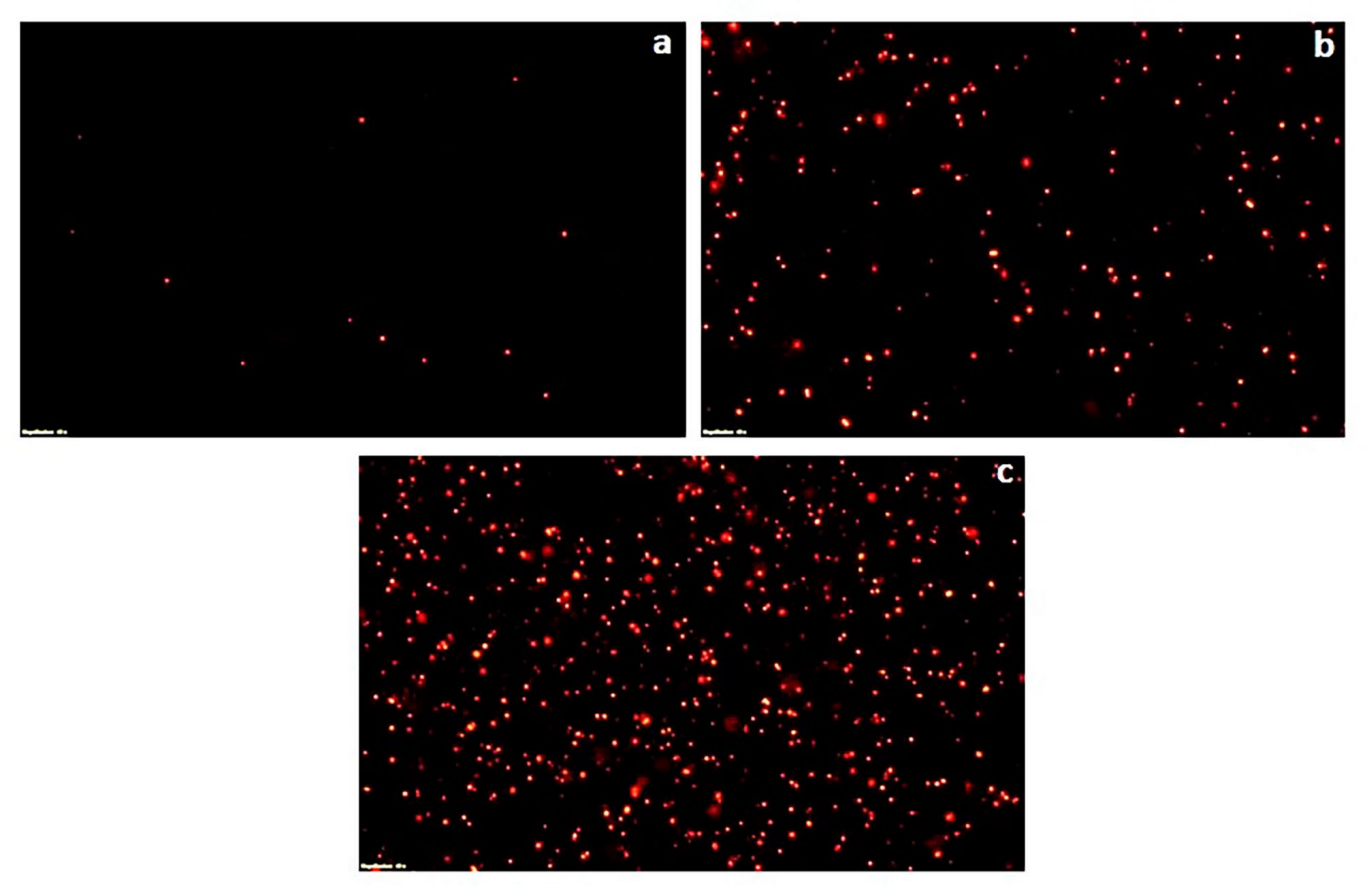
Fluorescent microscopic images of propidium iodide-stained *S. commune* (Sch1) extract treated/untreated *V. cholerae* under 40X. **(a)** untreated; **(b)**  *S. commune* (Sch1) extract treated cells after 2 h **(c)**  *S. commune* (Sch1) extract treated cells after 4 h

**Fig. 4:**
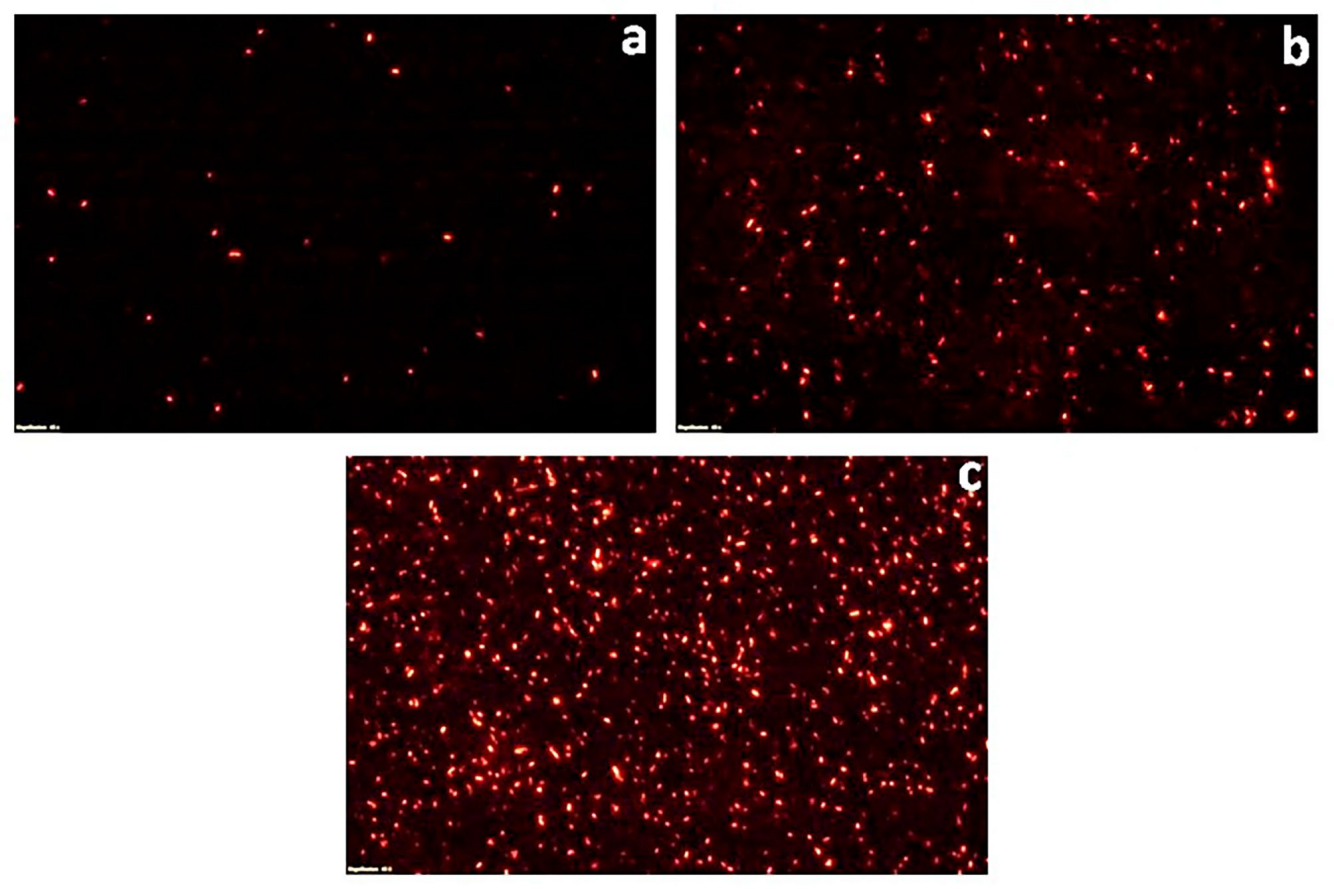
Fluorescent microscopic images of propidium iodide-stained *S. commune* (Sch1) extract treated/untreated *P. aeruginosa* under 40X. **(a)** untreated; **(b)**  *S. commune* (Sch1) extract treated cells after 3 h **(c)**  *S. commune* (Sch1) extract treated cells after 6 h

**Fig. 5:**
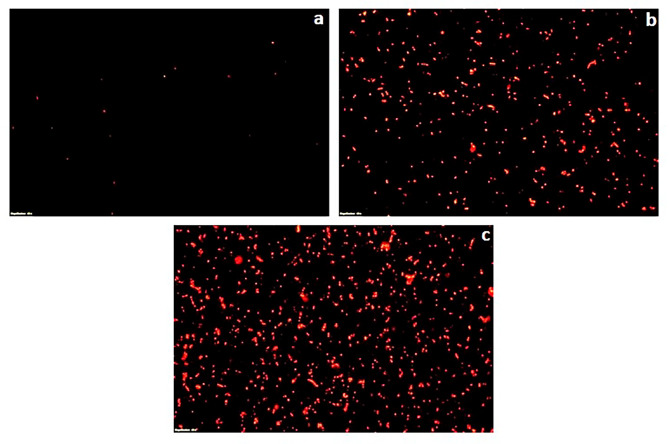
Fluorescent microscopic images of propidium iodide-stained *S. commune* (Sch1) extract treated/untreated *S. enterica* under 40X. **(a)** untreated; **(b)**  *S. commune* (Sch1) extract treated cells after 30 min; **(c)**  *S. commune* (Sch1) extract treated cells after 1 h

**Fig. 6:**
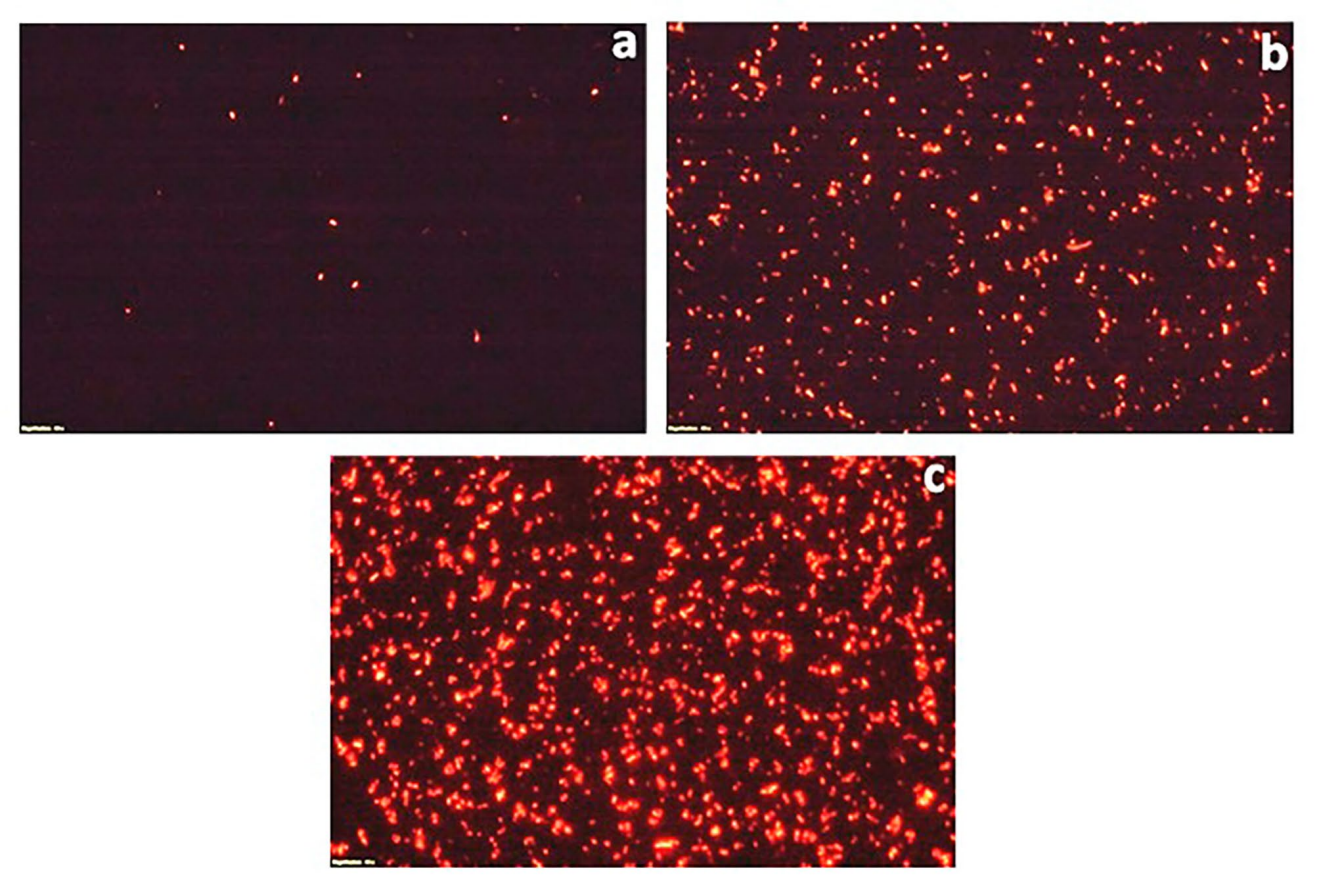
Fluorescent microscopic images of propidium iodide-stained *S. commune* (Sch1) extract treated/untreated *K. pneumoniae* under 40X. **(a)** untreated; **(b)**  *S. commune* (Sch1) extract treated cells after 1 h; **(c)**  *S. commune* (Sch1) extract treated cells after 2 h

**Fig. 7:**
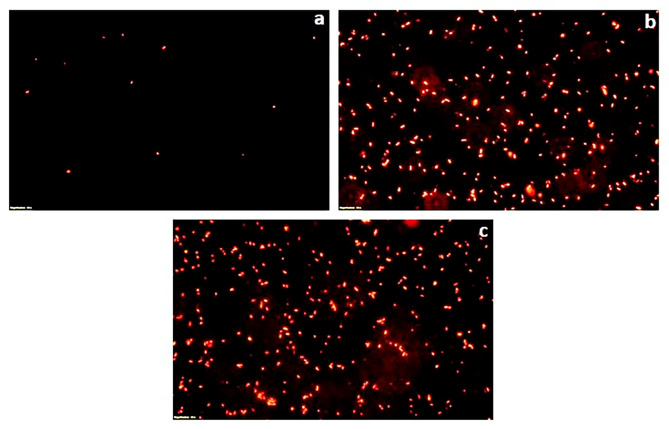
Fluorescent microscopic images of propidium iodide-stained *S. commune* (Sch1) extract treated/untreated *E. coli* under 40X. **(a)** untreated; **(b)**  *S. commune* (Sch1) extract treated cells after 2 h; **(c)**  *S. commune* (Sch1) extract treated cells after 4 h

To further validate the bactericidal mode of action of *S. commune* (Sch1) extract, the treated bacterial cells were stained with propidium iodide at different time intervals and visualised under fluorescent microscope (Figs. 2, 3, 4, 5, 6 and 7). Propidium iodide can only enter the bacterial cell after the cell membrane is compromised. Time dependent increase in the number of propidium iodide-stained cells was observed in all the *S. commune* (Sch1) extract treated bacteria, indicating the killing of the treated bacterial cells, with increase in incubation time.

### Biofilm inhibitory activity

Biofilm formation by pathogenic bacteria poses a serious health threat because biofilm forming bacteria survive under unfavourable conditions, such as the presence of antibiotics, host immune system and a number of external stresses. The resistant properties of the biofilm are the main reason behind the persistent chronic infection and antimicrobial resistance.

**Fig. 8:**
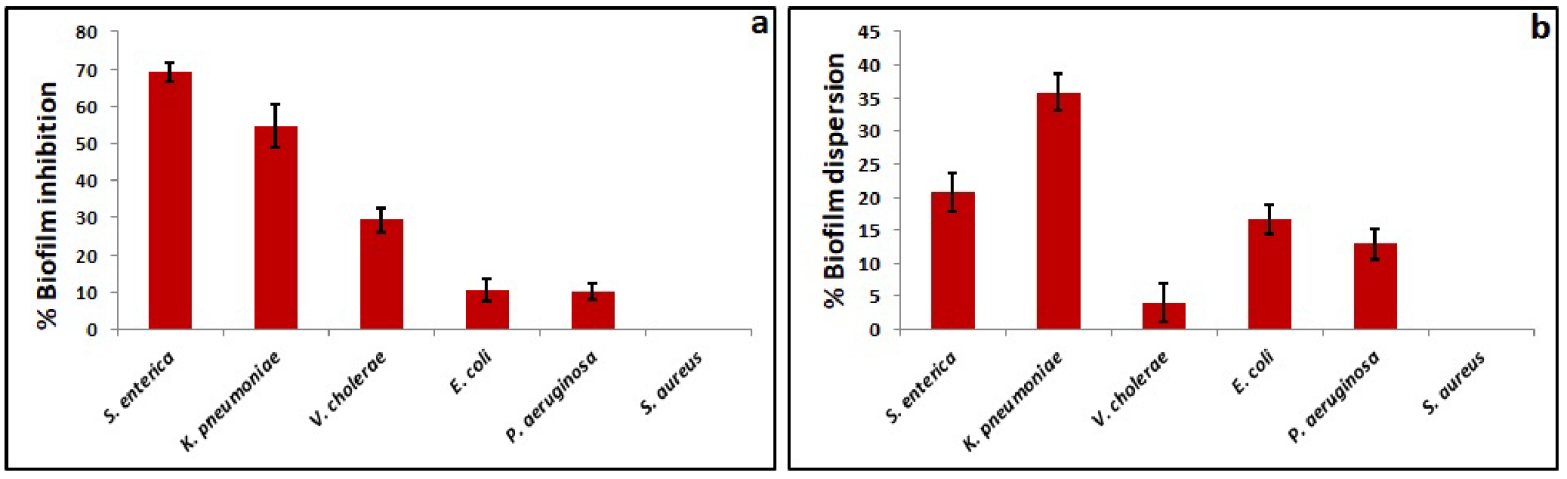
Biofilm inhibitory and biofilm dispersion potential of *S. commune* (Sch1) extract against various bacterial pathogens

In the present study, biofilm inhibitory potential of *S. commune* (Sch1) extract at sub-MIC values was assessed against different bacterial pathogens. *S. commune* (Sch1) extract exhibited good biofilm inhibitory potential against *S. enterica* (69.30 ± 2.54%) and *K. pneumoniae* (54.69 ± 5.97%) at sub-MIC values. On the other hand, *S. commune* (Sch1) extract showed moderate inhibitory activity against biofilm of *V. cholerae* (29.40 ± 3.32%). In case of *E. coli* (10.69 ± 3.10%) and *P. aeruginosa* (10.19 ± 2.29%) low biofilm inhibition was observed. *S. commune* (Sch1) extract showed no biofilm inhibitory activity against *S. aureus* at sub-MIC value (Fig. 8a).

**Fig. 9:**
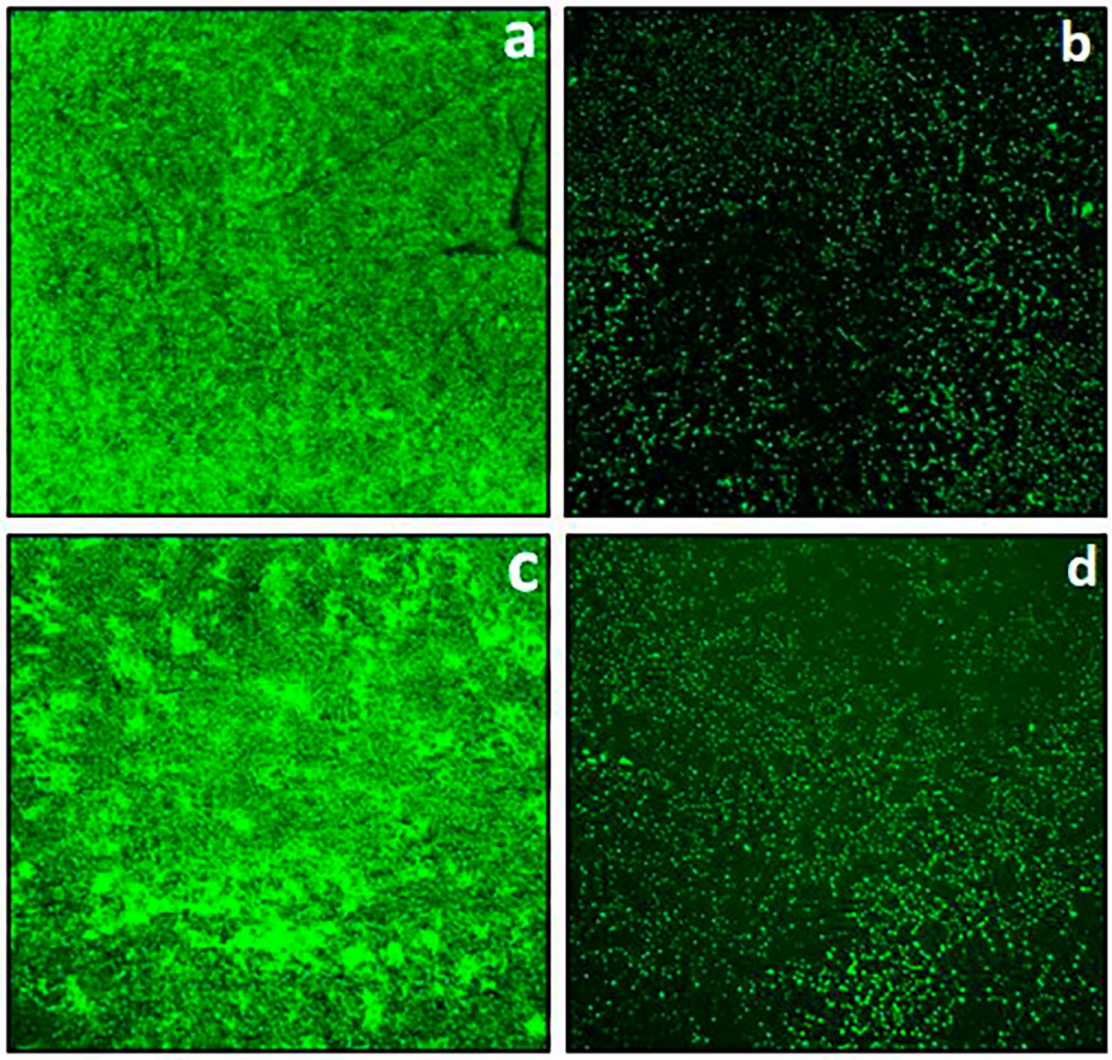
Fluorescent microscopic images of acridine orange stained biofilms of *S. enterica* and *K. pneumoniae*. **(a)**  *S. enterica* untreated cells; **(b)**  *S. enterica* cells treated with sub-MIC of *S. commune* (Sch1) extract; **(c)***K. pneumoniae* untreated cells; **(d)**  *K. pneumoniae* cells treated with sub-MIC of *S. commune* (Sch1) extract

To further validate the biofilm inhibitory potential of *S. commune* (Sch1) extract fluorescent microscopic studies were done by staining the live bacteria with fluorescent dye acridine orange. Biofilms of *S. enterica* and *K. pneumoniae* were allowed to form in the presence or absence of *S. commune* (Sch1) extract and after 48 h the developed biofilms were stained with acridine orange. As shown in Fig. 9a and c, untreated *S. enterica* and *K. pneumoniae* developed thick biofilms, whereas in the presence of sub-MIC values of *S. commune* (Sch1) extract thin biofilms were formed (Fig. 9b and d).

### Effect of ***S. commune*** (Sch1) extract on preformed biofilms

The effect of *S. commune* (Sch1) extract on the preformed biofilms of the bacterial pathogens was also studied. As shown in Fig. 8b, at sub-MIC value (5 mg/ml), *S. commune* (Sch1) extract caused 35.82 ± 2.76% dispersion of preformed biofilm of *K. pneumoniae*, but showed low biofilm dispersion activity against all the other bacterial pathogens.

## Discussion

Antibiotics are generally considered as magic bullets, as they possess the ability to target pathogenic microorganisms selectively without affecting the host. In the past 60 years, millions of metric tonnes of these antibiotics have been generated and employed for various purposes (Davies and Davies 2010; Zaman et al. 2017). Resistance to different antibiotics has been reported in various bacteria over the period of time. During the last 20 years, multidrug resistance in different pathogenic bacteria has risen to a level of pandemic resulting in millions of deaths (Kaur et al. 2021). Keeping in view the importance of search for novel antimicrobial agents, this study assessed the antibacterial, biofilm inhibition and biofilm dispersion potential of *S. commune* (Sch1) against various human pathogenic bacterial strains. Although some of the studies have reported the antibacterial potential of extracts derived from the fruiting bodies and mycelium of *S. commune* (Tripathi and Tiwary 2013; Appiah et al. 2017; Deka et al. 2017; Chen et al. 2021), detailed investigations on antibacterial and anti-biofilm activity are lacking.

*S. commune* (Sch1) extract was assessed for its antimicrobial activity against *S. aureus*, *K. pneumoniae*, *E.coli*, *P. aeruginosa*, *S. enterica* and *V. cholerae*. These bacterial strains have been associated with several severe illnesses. *S. aureus* a Gram positive bacteria belonging to phylum Firmicutes, is reported to show resistance against several antibiotics and is responsible for several diseases such as endocarditis, bacteremia-sepsis, pneumonia, osteomyelitis, skin diseases and arthritis (Lowy 2003; Tong et al. 2015; Dayan et al. 2016). *P. aeruginosa, E. coli, V. cholerae, S. enterica* and *K. pneumoniae* are Gram negative bacteria, which are well known for their association with severe to fatal diseases, including urinary tract infections, pneumonia, septicaemia, gastroenteritis, enteric fever, diarrhea, enteritis, neonatal meningitis etc. (Johnson and Stell 2000; Wolf and Elsässer-Beile 2009; Allocati et al. 2013; Almagro-Moreno and Taylor 2013; Eng et al. 2015; Navon-Venezia et al. 2017). Preliminary screening of *S. commune* (Sch1) extract showed good activity against these pathogenic bacteria. Detailed studies were conducted to determine minimum inhibitory concentration (MIC) and minimum bactericidal concentration (MBC). Lowest concentration of extract or compound which prevents the visible growth of the microorganisms followed by overnight incubation is known as MIC. This method is considered as “gold standard” to determine the susceptibility of microorganisms to antimicrobial compounds. MBC is the lowest concentration of extract or compound which inhibits the growth of any microorganism when sub-cultured on medium containing no extract or antimicrobial compound (Andrews 2001). In the present study, the MIC and MBC of *S. commune* (Sch1) extract against different pathogenic bacteria ranged between 1.25 and 10 mg/ml. Antimicrobial agents are considered as bacteriostatic if MBC/MIC is > 4 and bactericidal if MBC/MIC is ≤ 4 (Keepers et al. 2014; Mogana et al. 2020). In our study, the calculated MBC/MIC ratio was ≤ 2 against all the pathogens, hence, *S. commune* (Sch1) extract was considered as bactericidal.

Another commonly used strategy to determine the bacteriostatic or bactericidal nature of the antimicrobial compounds is time kill studies. Antimicrobial compound is considered as bactericidal if a decrease of ≥ 3 log_10_ CFU/ml is observed in treated sample as compared to initial inoculum, whereas reduction of < 3 log_10_ CFU/ml signifies bacteriostatic nature of compound (Kalia et al. 2009). The bactericidal nature of the extract was also confirmed in time kill studies, as the treated cells showed a gradual decline in viable cell count, with an increase in incubation time. Complete killing against all pathogens was recorded between 1 and 6 h, except in case of *S. aureus*, where more than 3 log_10_ CFU/ml decrease in viable cell count was observed after 8 h of incubation.

The findings of time kill assay were also validated using fluorescent microscopic observations. Bacterial cells were stained with propidium iodide, which is a fluorescent DNA stain and can enter the cell only after the membrane is compromised (Crowley et al. 2016). The number of propidium iodide stained cells after treatment with *S. commune* (Sch1) extract was found to increase with an increase in incubation time, in all the tested bacterial pathogens. These studies also confirmed the bactericidal nature of the extract.

As mentioned previously, biofilms are the microbial cells enclosed in self synthesized matrix, which are responsible for chronic bacterial infections. Biofilm formation occurs in a series of steps including reversible attachment, irreversible attachment, microcolony formation, maturation, and lastly dispersion of the biofilm. Biofilms are responsible for both tissue and device related infections such as rhinosinusitis, cystic fibrosis, periodontitis, osteomyelitis, endocarditis, meningitis, non-healing chronic wounds, prosthesis and kidney infections (Khatoon et al. 2018; Rather et al. 2021). Thus, compounds possessing both antibacterial and anti-biofilm activities could be a better approach for controlling the infections. In our study, biofilm inhibitory potential of *S. commune* (Sch1) extract was evaluated against *S. aureus*, *K. pneumoniae*, *E. coli*, *P. aeruginosa*, *S. enterica*, and *V. cholerae* at sub-MIC values. All these pathogenic bacterial strains have been reported to form biofilms and to cause several latent, acute and chronic diseases (Harrell et al. 2021; Schulze et al. 2021; Rather et al. 2021). *S. commune* (Sch1) extract showed good biofilm inhibitory potential against *K. pneumoniae* and *S. enterica*. To validate the biofilm inhibitory activity of *S. commune* (Sch1) extract against *K. pneumoniae* and *S. enterica*, fluorescent microscopy of the formed biofilm of treated and untreated cells was done. Fluorescence is directly proportional to formed biofilm, thicker the biofilm; higher the fluorescence. In the present study decreased fluorescence was observed in *S. commune* (Sch1) extract treated cells in comparison with untreated control, indicating biofilm inhibitory activity. This is the first report revealing the biofilm inhibitory activity of an endophytic *S. commune*.

Another important feature expected in an ideal biofilm inhibitory compound is to disperse the pre-existing biofilms. Disruption of the pre-existing biofilms is very difficult because of the restricted exposure of the antimicrobials to the microbial cells present in the EPS matrix. In addition to this, nutritional scarcity, slow growth, persister cell formation and adaptive stress response also forms a multi-layered defence system (Stewart 2002). In the present study, biofilm dispersion potential of *S. commune* (Sch1) extract was also assessed against all the tested pathogens. *S. commune* (Sch1) extract showed moderate biofilm dispersion activity against *K. pneumoniae.* Weak biofilm dispersion potential was observed against all the other bacterial pathogens. The observed % dispersion was lower than the % inhibition which could be due to the resistant and impermeable nature of the pre-existed biofilms.

The observed activities could be due to the presence of phenolics and terpenoids in *S. commune* (Sch1) extract, revealed in a previous study (Sharma et al. 2021). Phenolics and terpenoids have also been reported to exhibit good antimicrobial activity (Maddox et al. 2010; Rahman et al. 2014; Tyagi et al. 2015; Guimarães et al. 2019; Achika et al. 2020). Alves et al. (2013) documented the antimicrobial activity of various phenolic compounds present in different mushrooms. In another study, terpenoids derived from *Trichodesma amplexicaule* have been reported to possess good antimicrobial activity (Singh and Singh 2003). Different modes of action are exhibited by phenolics and terpenoids for antimicrobial activity. These mechanisms include inhibition of gene expression, inhibition of vital enzymes and other virulence factors, damage to cell membrane etc. (Miklasińska-Majdanik et al. 2018; Sumayya et al. 2020; Yang et al. 2021). Phenolics and terpenoids are reprted to show good biofilm inhibitory activity in some other studies also (Raut et al. 2013; Luís et al. 2014).

*S. commune* (Sch1) extract showed good antibacterial and biofilm inhibitory activity against various pathogenic bacteria. Results obtained in the present study showed that it can be exploited in the field of antibacterial therapeutics as a source of effective bioactive compounds. This is the first study revealing the biofilm inhibitory potential of endophytic *S. commune*.

## Data Availability

All data generated or analysed during this study are included in this published article.

## Abbreviations

EPS: Extracellular polymeric substances
NFCCI: National Fungal Culture Collection of India
NCIM: National Collection of Industrial Microorganisms
MTCC: Microbial Type Culture Collection
MIC: Minimum inhibitory concentration
MBC: Minimum bactericidal concentration
CFU: Colony-forming unit
PBS: Phosphate buffer saline
